# Potential of resveratrol in enrichment of neural progenitor-like cell induction of human stem cells from apical papilla

**DOI:** 10.1186/s13287-020-02069-9

**Published:** 2020-12-14

**Authors:** Anupong Songsaad, Thanasup Gonmanee, Nisarat Ruangsawasdi, Chareerut Phruksaniyom, Charoensri Thonabulsombat

**Affiliations:** 1 Department of Anatomy, Faculty of Science, Mahidol University, 272 RAMA VI Road, Ratchathewi, Bangkok, 10400 Thailand; 2 Chakri Naruebodindra Medical Institute, Faculty of Medicine Ramathibodi Hospital, Mahidol University, 111 Bang Pla, Bang Phli, Samut Prakan, 10540 Thailand; 3 Department of Pharmacology, Faculty of Dentistry, Mahidol University, 6 Yothi Road, Ratchathewi, Bangkok, 10400 Thailand

**Keywords:** Resveratrol, Neural progenitor-like cells, Human stem cells from apical papilla

## Abstract

**Introduction:**

Stem cell transplantation of exogenous neural progenitor cells (NPCs) derived from mesenchymal stem cells (MSCs) has emerged as a promising approach for neurodegenerative disease. Human stem cells from apical papilla (hSCAPs) are derived from migratory neural crest stem cells and exhibit a potential of neuronal differentiation. However, their neuronal differentiation is low and unpredictable. Resveratrol has been described as a sirtuin 1 (*SIRT1*) activator which plays an important role in enhancing neuronal differentiation. In this study, we investigate the potential of resveratrol as an enhancer on neuronal differentiation through NPCs induction of hSCAPs.

**Methods:**

Stem cells were isolated from human apical papilla and characterized as MSCs. The cellular toxicity of resveratrol treatment to the characterized hSCAPs was investigated by MTT assay. The non-cellular toxicity concentrations of resveratrol were assessed with various pre-treatment times to select the optimal condition that highly expressed the neural progenitor gene, *NES*. Consequently, the optimal condition of resveratrol pre-treatment was synergistically performed with a neuronal induction medium to trigger neuronal differentiation. The differentiated cells were visualized, the genes profiling was quantified, and the percentage of neuronal differentiation was calculated. Moreover, the intracellular calcium oscillation was demonstrated.

**Results:**

The cellular toxicity of resveratrol was not observed for up to 50 μM for 12 h. Interestingly, hSCAPs pre-treated with 10 μM resveratrol for 12 h (RSV-hSCAPs) significantly expressed *NES*, which is determined as the optimal condition. Under neuronal induction, both of hSCAPs and RSV-hSCAPs were differentiated (d-hSCAPs and RSV-d-hSCAPs) as they exhibited neuronal-like appearances with Nissl substance staining. The highest expression of *NES* and *SOX1* was observed in RSV-d-hSCAPs. Additionally, the percentage of neuronal differentiation of RSV-d-hSCAPs was significantly higher than d-hSCAPs for 4 times. Importantly, the neuronal-like cells exhibited slightly increasing pattern of calcium intensity.

**Conclusion:**

This study demonstrated that pre-treatment of resveratrol strongly induces neural progenitor marker gene expression which synergistically enhances neural progenitor-like cells’ induction with neuronal induction medium.

## Introduction

The neurological disorders of the central nervous system (CNS) account for more than 10% of death and new causes of permanent disability. The most common neurological disorders of CNS consist of Parkinson’s disease, Alzheimer’s disease, stroke, and traumatic brain injuries [1]. The cardinal characteristic that these neurological disorders have been defined by is loss of neurons and the corresponding loss of function and disabilities [2]. Adult neurogenesis is the process of generating new neurons from NPCs [3]. Unfortunately, endogenous repairing of affected CNS via the NPCs is restricted and limited [4]. To improve the quality of life of patients who are suffering from neurological disorders, a replacement of degenerated neurons with exogenous NPCs could be a potential treatment to regenerate the damaged CNS.

Human stem cells from apical papilla were discovered by Sonoyama W. in 2008 [5]. As the name implies, the hSCAPs are localized at the apex part (apical papilla) of the developing tooth which contains the stem cells and have characterized as MSCs [6]. According to the origin, the hSCAPs represent early stem cell populations that exhibit superior stem cell properties, including self-renewal and differentiation potency, to the other dental-derived stem cells (DSCs), which are isolated from a mature tissue [7]. MSCs can be characterized by differentiation into at least 3 specialized lineages: adipocytes, osteocytes, and chondrocytes [8]. Moreover, neuronal cells can be generated from MSCs by administration of extrinsic factors in the neuronal induction medium as demonstrated in several MSCs-derived tissues, including the adipose tissue [9], bone marrow [10], umbilical cord [11], cord blood [12], periodontal ligament [13], and both deciduous and permanent teeth [14]. Therefore, MSCs are an efficient stem cell source for neuronal differentiation. However, the ability of neuronal differentiation of MSCs has some limitations involving a low percentage of differentiation and unpredictability of differentiated cell type [15]. Moreover, most of the engrafted cells die within a week of transplantation, and only a few engrafted cells successfully integrated into the injured area [16].

Recently, medicinal plant-derived natural compounds have become of interest as alternative sources of new therapeutic agents for neurodegenerative disease. Moreover, they exert their potential effects by enhancing neuronal differentiation and adult neurogenesis [17]. Resveratrol (3,4′,5-trihydroxy-trans-stilbene) is defined as a natural non-flavonoid polyphenol compound with a stilbene structure obtained from various plants including grapes, peanuts, pine trees, and berry plants [18]. Remarkably, resveratrol has been identified as a *SIRT1* activator playing an important role in enhancing neuronal differentiation and neuroprotection [19]. Previous studies have demonstrated the potential of *SIRT1* activator resveratrol in inducing neuronal differentiation and structural morphological change of MSCs derived from the bone marrow [20], umbilical cord [21], cord blood [12], and dental pulp [15] into the neuronal cells. Also, pre-treatment of resveratrol to MSCs at an optimal condition significantly promotes NPCs gene expression [20]. Despite recent progress, enhancing NPCs induction of hSCAPs by resveratrol has not yet been investigated.

In this study, we demonstrated the potential effect of resveratrol on neuronal differentiation using the optimal condition that directly drives neuronal differentiation into neural progenitor-like cells of hSCAPs.

## Methods

### Tooth sample collection

Human impacted third molars (*n* = 7) were collected from Thai patients (15–20 years) at the Faculty of Dentistry, Mahidol University, Thailand. The ethical consideration and research protocol were approved by the Ethics Committee on Human Rights Related to Human Experimentation of Faculty of Dentistry/Faculty of Pharmacy, Mahidol University (COE. No. MU-DT/PY-IRB 2019/027.2405). The inclusion criteria of the teeth consist of the presence of apical papilla tissue, caries-free, and no sign of pulp necrosis, trauma, or periodontal disease.

### Cell isolation and culture

The isolation of hSCAPs was performed by the enzymatic-disaggregation method as previously described [6]. Briefly, the teeth were collected in a proliferating medium consisting of Alpha Minimum Essential Medium (αMEM, Gibco, Life Technologies, Grand Island, NY, USA), supplemented with 10% fetal bovine serum (FBS, Gibco, Life Technologies), 100 U/mL penicillin, and 100 μM/mL streptomycin (Gibco, Life Technologies), and washed with 0.1 M phosphate buffer saline (PBS, Sigma-Aldrich, St. Louis, MO, USA). Following teeth extraction, the apical papilla tissue was separated, dissected into smaller pieces, and digested with a cocktail of 3 mg/mL collagenase type I (Worthington, Lakewood, NJ, USA) and 4 mg/mL dispase II (Sigma-Aldrich) at 37 °C for 1 h. The digested tissue was filtered through 70 μm cell strainer (Falcon™, Fisher Scientific, Waltham, MA, USA), seeded into a cell culture vessel (T-75 cm^2^ flask, Nunc™, Thermo Scientific, Waltham, MA, USA), and cultured in the proliferating medium at 37 °C, 5% CO_2_, and 95% humidity incubator. The medium was changed every 2 days until confluence was achieved. Then, the cells were subculture to expand the cell population. Cells at passages 2–6 were used in this study.

### Cell surface molecule marker analysis

The uncharacterized cells (1 × 10^6^ cells) were harvested and the cell surface antigen molecules were analyzed by BD FACS Canto Flow cytometer (BD Biosciences, San Jose, CA, USA). The cells were detected for MSCs specific markers using antibodies as follows: anti-human CD73 (APC/Cy7) (Biolegend, San Diego, CA, USA), anti-human CD90 (PE) (Biolegend), anti-human CD105 (Alexa Flour® 488) (Biolegend), and anti-human CD146 (PerCP/Cy5.5) (Biolegend). An antibody against hematopoietic stem cell marker, anti-human CD34 (APC) (Biolegend), was used as a negative control. The level of cell surface antigen molecules expression was analyzed using the BD FACSDiva™ software (BD Biosciences).

### Colony-forming unit fibroblast

The uncharacterized cells were seeded in triplicate into 6-well plates (Nunc™_,_ Thermo Scientific) at a density of 500 cells/well and cultured in the proliferating medium for 12 days. The medium was changed every 2 days. The colonies of these cells were visualized by Giemsa staining and captured by the Compact Cell Culture Microscope, CKX3 (Olympus, Hamburg, Germany).

### Osteogenic differentiation

The uncharacterized cells were seeded in 24-well plates (Nunc™, Thermo Scientific) at a density of 2 × 10^4^ cells/well and cultured in the proliferating medium until reaching 80% confluence. Osteogenic differentiation was induced by culturing for 4 weeks in an osteogenic induction medium consisting of 0.1 μM dexamethasone (Sigma-Aldrich), 50 μg/mL ascorbate-2-phosphate (Sigma-Aldrich), 10 mM β-glycerophosphate (Sigma-Aldrich) in αMEM, 10% FBS, 100 U/mL penicillin, and 100 μM/mL streptomycin. The medium was changed every 2 days. The calcification of an extracellular matrix was observed with Alizarin red staining and captured by the Compact Cell Culture Microscope, CKX3 (Olympus).

### Adipogenic differentiation

The uncharacterized cells were seeded in 24-well plates (Nunc™, Thermo Scientific) at a density of 2 × 10^4^ cells/well and cultured in the proliferating medium until reaching 100% confluence. Adipogenic differentiation was induced by culturing for 6 weeks in an adipogenic induction medium consisting of the proliferating medium supplemented with 1 μM dexamethasone (Sigma-Aldrich), 50 μM indomethacin (Sigma-Aldrich), 1 μg/mL insulin (Sigma-Aldrich), and 0.5 mM 3-isobutyl-1-methylxanthine (IBMX, Sigma-Aldrich). Oil Red O was stained to visualize lipid droplets and captured by the Compact Cell Culture Microscope, CKX3 (Olympus).

### Cell viability of resveratrol-treated hSCAPs

The cell viability of resveratrol-treated hSCAPs was performed by the methylthiazolyldiphenyl-tetrazolium bromide (MTT, Sigma-Aldrich) assay. Resveratrol (trans-3, 4′, 5-trihydroxystibene; Sigma-Aldrich) was freshly prepared as a 100 μM stock solution by diluting with αMEM, 100 U/mL penicillin, 100 μM/mL streptomycin, and maintained in dark condition. The characterized hSCAPs were seeded in 96-well plates (Nunc™, Thermo Scientific) at a density of 1 × 10^4^ cells/well. After 24 h, the hSCAPs were treated with different concentrations of resveratrol (0, 5, 10, 15, 25, 50, and 100 μM) for 6, 12, and 24 h. Then, the MTT assay was performed. The MTT working solution (0.5 mg/mL) was added, and the plates were incubated for an additional 2 h at 37 °C. After centrifugation, the solution was replaced with dimethyl sulfoxide (DMSO, Fisher Scientific). The absorbance of each well at 570 nm and 690 nm was measured with a micro-plate reader (Epoch, Fisher Scientific, Waltham, MA, USA). The percentage of cell viability of hSCAPs in resveratrol treatments (A_570_-A_690_ of experimental group ×  100/A_570_-A_690_ of control group) (*n* = 5) and the 50% inhibitory concentration (IC_50_) of resveratrol pre-treatment on hSCAPs were reported.

### Optimal condition of resveratrol pre-treatment

The hSCAPs were seeded in 6-well plates at a density 1 × 10^5^ cells/well. After 24 h, the cells were incubated with different non-cellular toxicity concentrations of resveratrol for 12 h, and qRT-PCR was performed to select the concentration of resveratrol that induced the highest *NES* expression of hSCAPs. Then, the concentration was used to assess *NES* expression at various incubation times (1, 6, 12, and 24 h). The treatments were also investigated for morphological change with β-III tubulin immunocytochemistry staining. The hSCAPs treated with resveratrol at the concentration and incubation time that brought the highest *NES* expression will be termed “RSV-hSCAPs.” The RSV-hSCAPs were validated the neuronal genes profiling with *SOX1*, *PAX6*, and immunofluorescences with Ki67, neurofilaments (NF), and were further induced into neuronal differentiation.

### Neuronal induction

The hSCAPs were seeded on poly-d-lysine (Sigma-Aldrich) coated cover slips (Electron Microscopy Sciences, Hatfield, PA, USA) in 6-well plates at a density 1 × 10^5^ cells/well and pre-incubated with the optimal condition of resveratrol (RSV-hSCAPs) or without resveratrol (hSCAPs). Then, both hSCAPs and RSV-hSCAPs were exposed to 2 phases of neuronal induction medium. First, the cells were incubated with Dulbecco’s Modified Eagle Medium: Nutrient Mixture F-12 (Ham) (DMEM/F-12, Gibco, Life Technologies) supplemented with 10% FBS, 100 U/mL penicillin, 100 μM/mL streptomycin, 10 ng/mL basic fibroblast growth factor (bFGF, Gibco Life Technologies), and 500 μM β-mercaptoethanol (Sigma-Aldrich) for 24 h. After that, the cells were induced into a phase II neuronal induction medium which consisted of DMEM/F-12, 100 U/mL penicillin, 100 μM/mL streptomycin, 2% DMSO, and 100 μM butylated hydroxyanisole (BHA, Sigma-Aldrich) for 6 h. The negative control hSCAPs (crt-hSCAPs) was pre-incubated for 12 h with αMEM, 100 U/mL penicillin, 100 μM/mL streptomycin, and then cultured with DMEM/F-12, 10% FBS, 100 U/mL penicillin, and 100 μM/mL streptomycin for 24 h. The medium was then replaced with DMEM/F-12, 100 U/mL penicillin, and 100 μM/mL streptomycin for 6 h.

### Immunocytochemistry

The specimens were fixed in 4% paraformaldehyde (Sigma-Aldrich) in PBS at room temperature for 1 h, followed by 20% ice-cold methanol (Sigma-Aldrich) in PBS for 5 min, and then washed with PBS. Subsequently, the specimens were permeabilized with 0.5% Triton X-100 (Sigma-Aldrich) in PBS overnight at 4 °C and blocked with 15% bovine serum albumin (BSA, Sigma-Aldrich) at 4 °C for 12 h. The specimens were incubated overnight at 4 °C with anti-mouse Nestin antibody (Biolegend) at a dilution of 1: 500, anti-mouse β-III tubulin antibody (Biolegend) at a dilution of 1: 1000, anti-mouse Ki67 (Developmental Studies Hybridoma bank, Iowa City, IA, USA) at a dilution 1:100, and anti-mouse NF (Developmental Studies Hybridoma bank) at a dilution 1:100 which diluted with 5% BSA in PBS with 0.05% Tween-20 (Sigma-Aldrich). Then, the specimens were conjugated with goat anti-mouse IgG highly cross-adsorbed secondary antibody, Alexa Fluor plus 488 (Invitrogen, New York, NY, USA) at a dilution of 1: 1000 at room temperature for 4 h. Nuclei were counterstained and mounted with ProLong™ Diamond Antifade Mountant with DAPI (Invitrogen). The samples were visualized and captured by the Digital Fluorescence Microscope, BX53 (Olympus). The percentage of neuronal differentiation (the number of differentiated cells × 100/total cells) was quantified using the ImageJ program (NIH, Bethesda, MD, USA) by random counting (*n* = 5).

### Quantitative real-time reverse transcription polymerase chain reaction (qRT-PCR)

Total RNA was extracted using the Nucleospin RNA plus kit (Macherey-Nagel, Bethlehem, PA, USA) and converted into cDNA using iScript RT Supermix (Bio-Rad, Hercules, CA, USA). The qRT-PCR was performed using KAPA SYBR® FAST qPCR kits (Sigma-Aldrich) with CFX96™ real-time PCR detection system (Bio-Rad). The qRT-PCR reaction conditions were 95 °C for 3 min, followed by 40 cycles of 95 °C for 3 s and 52 °C for 30 s. The interesting primers (Integrated DNA Technologies, Gemini Singapore Science Park II, Singapore) used in this study are listed in Table 1. The glyceraldehyde 3-phosphate dehydrogenase (*GAPDH*) was used as an internal control, and the expression of interested genes was measured by 2^−ΔΔCt^ method [22].

**Table 1:** Forward and reverse primers for qRT-PCR

Genes	Primers	Sequences (5′-3′)	References
*NES*	Forward	CTGCTACCCTTGAGACACCTG	NM_006617.1
	Reverse	GGGCTCTGATCTCTGCATCTAC	
*MAP-2*	Forward	CGAAGCGCCAATGGATTCC	NM_001039538.1
	Reverse	TGAACTATCCTTGCAGACACCT	
*TUBB3*	Forward	GGCCAAGGGTCACTACACG	NM_006086.3
	Reverse	GCAGTCGCAGTTTTCACACTC	
*SOX1*	Forward	GTAAGGGAACCCGGGGAATG	NM_005986.3
	Reverse	GGGGTCTTCCCTTCCTCCT	
*PAX6*	Forward	AACAGACACAGCCCTCACAAACA	NM_001368892.2
	Reverse	CGGGAACTTGAACTGGAACTGAC	
*GAPDH*	Forward	CTGGGCTACACTGAGCACC	NM_001256799
	Reverse	AAGTGGTCGTTGAGGGCAATG	

### Cresyl violet staining

The specimens were fixed in 4% paraformaldehyde at room temperature for 60 min, and then washed with PBS for 5 min and double distill water (ddH_2_O) for 1 min. Subsequently, the specimens were incubated with Cresyl Violet Acetate working solution (Electron Microscopy Sciences) at dark condition for 60 min. Then, the specimens were washed with ddH_2_O, followed by serial dehydration of 90%, 95%, and 100% ethanol respectively. Cell imaging was captured by the Compact Cell Culture Microscope, CKX3 (Olympus).

### Intracellular calcium oscillation

In order to identify potential of neuronal differentiation, we evaluated calcium influx which is an indicator for neurotransmitter transmission. The intracellular calcium assessment was described in previous study [23]. The specimens were incubated with 3 μM Fluo-3 AM (Invitrogen) and 0.08% pluronic acid (Invitrogen) in DMEM/F-12, 100 U/mL penicillin, and 100 μM/mL streptomycin at 37 °C for 60 min. Subsequently, the specimens were washed with DMEM/F-12, 100 U/mL penicillin, 100 μM/mL streptomycin, and PBS. The specimens were maintained in Tyrode’s solution (5 mM KCl, 129 mM NaCl, 2 mM CaCl_2_, 1 mM MgCl_2_, 30 mM glucose, and 25 mM HEPES, pH 7.4) (all from Sigma-Aldrich). The neurotransmitter releasing ability of differentiated cells was simulated with 50 mM KCI. The intensity of calcium was recorded time-lapse at excitation 506 nm for 3 min by the live-cell fluorescence microscope, IX83XDC (Olympus) and interpreted using the ImageJ program (NIH).

### Statistical analysis

The individual experiment was repeated 3 times. The data were expressed as the mean ± standard error of mean (SEM), the difference between the experimental groups and the control group were compared using Tukey’s Multiple Comparison Test via GraphPad Prism version 5.00.288 (San Diego, CA, USA). The differences with *p value < 0.05 and ***p value < 0.001 were considered significant.

## Results

### Characterization of hSCAPs

**Fig. 1:**
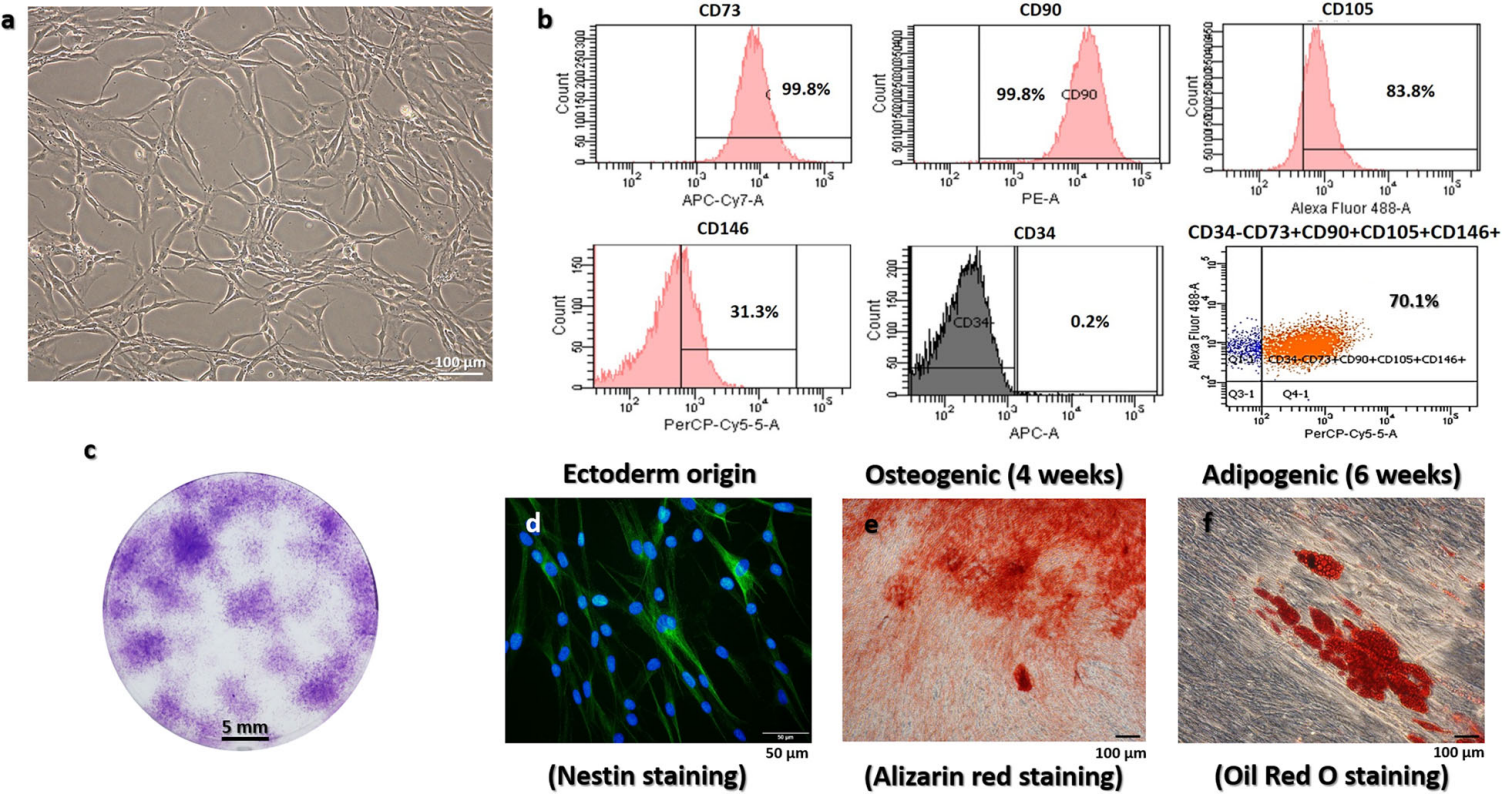
Characterization of hSCAPs. **a** Isolated cells present the typical fibroblast and spindle-like-shaped morphology. **b** The cells are positive for CD73, CD90, CD105, and CD146 but negative for CD34. The amount of isolated cells that expressed these markers (CD73+, CD90+, CD105+, CD146+, and CD34−) are highly expressed. **c** The cells efficiently form colonies. **d** The cells reveal the neural crest derivative origin with nesting staining. **e** Osteogenic differentiation was demonstrated with Alizarin red staining of calcified nodule. **f** Adipogenic differentiation was revealed with lipid droplets stained by Oil Red O. Scale bars: **a**, **e**, and **f** = 100 μm, **c** = 5 mm, and **d** = 50 μm

Firstly, the isolated cells from human apical papilla tissue presented the typical fibroblast and spindle-like shape morphology in plastic adherent culture (Fig. 1a). Secondly, the cell surface antigen molecule analysis via flow cytometry demonstrated the cells highly expressed MSCs markers, CD73 (99.8%), CD90 (99.8%), CD105 (83.8%), CD146 (31.3%), and negatively expressed CD34 (0.2%). The isolated cells that co-expressed CD73+, CD90+, CD105+, CD146+, and CD34− were a major population (70.1%) (Fig. 1b). The cells effectively formed colonies indicating the self-renewal ability (Fig. 1c). In evaluating the neural crest derivative origin, the immunofluorescence reveals the Nestin expression of the isolated cells (Fig. 1d). Multipotential differentiation properties were shown by osteogenic and adipogenic induction. The cells produced calcified nodules (Fig. 1e) and lipid droplets (Fig. 1f) as revealed by Alizarin red and Oil Red O staining, respectively. These indicated that the cells were able to differentiate into osteocytes and adipocytes. Taken together, the isolated cells exhibited MSCs properties, verified as hSCAPs.

### The cellular toxicity of resveratrol on hSCAPs

**Fig. 2:**
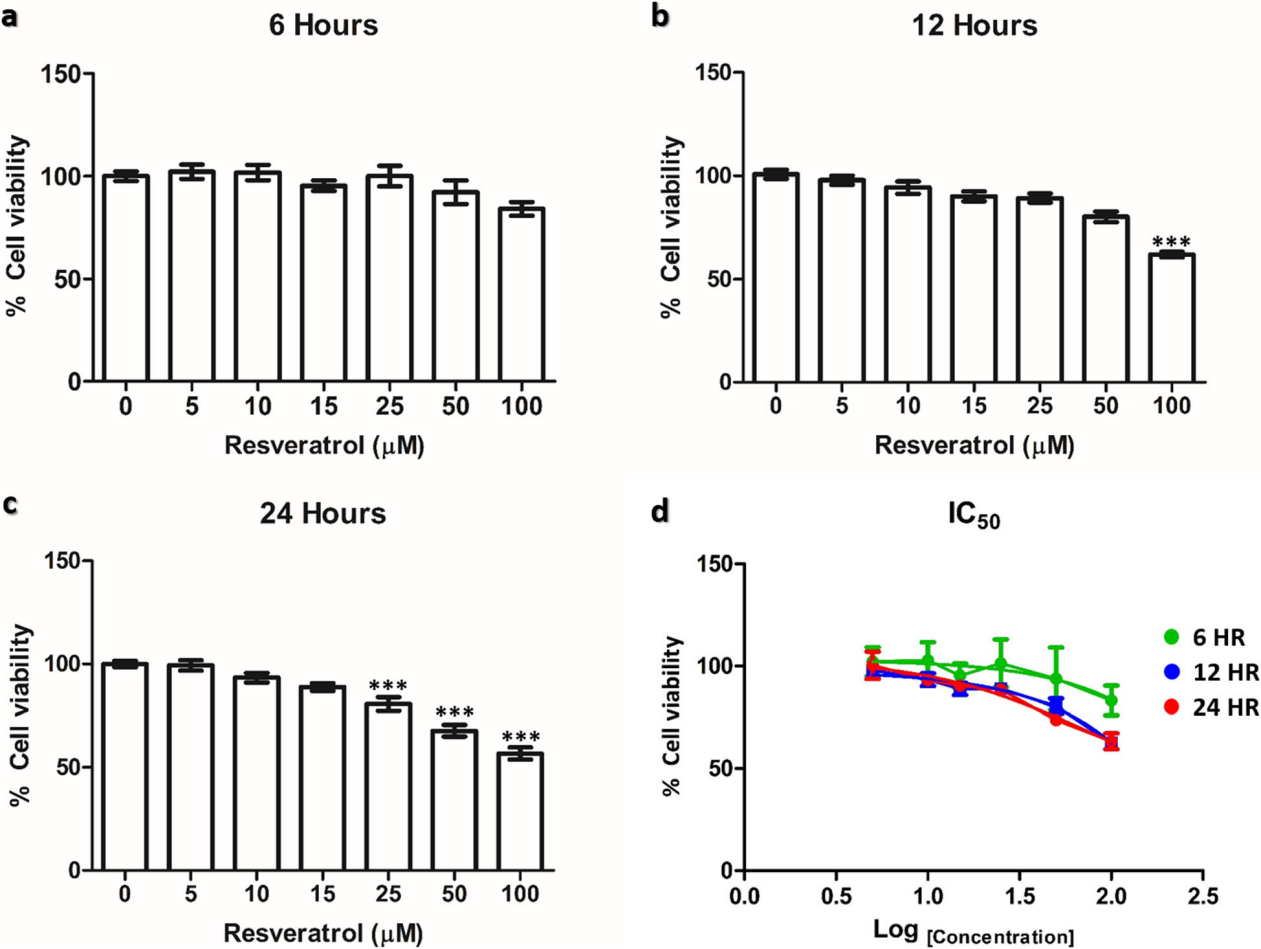
The cellular toxicity of RSV-hSCAPs. **a**–**c** The percentage of cell viability of RSV-hSCAPs during 0–100 μM of resveratrol for 6, 12, and 24 h, respectively. Cellular toxicity was not observed for up to 100 μM for 6 h, 50 μM for 12 h, and 15 μM for 24 h. **d** The IC_50_ of resveratrol treatment on hSCAPs for 6, 12, and 24 h. Data were expressed as the mean ± SEM; *n* = 3, ***p < 0.001

To evaluate the toxicity of resveratrol on hSCAP viability, the hSCAPs were incubated with 0–100 μM of resveratrol for 6, 12, and 24 h. The viability of the cells did not observe cytotoxicity at any concentrations of resveratrol treatment for 6 h (Fig. 2a). In contrast, the cellular viability significantly decreased at 12 h in 100 μM (Fig. 2b) and 24 h in 25 μM resveratrol treatment (Fig. 2c), compared to the control group. Additionally, the IC_50_ of resveratrol treatment on hSCAPs were shown as 3380 mM (6 h), 1501 μM (12 h), and 73.33 μM (24 h) (Fig. 2d). As a result, the concentration of resveratrol ranging to 0–50 μM for 12 h pre-treatment was chosen for use in the next experiment.

### Optimal condition of resveratrol pre-treatment

**Fig. 3:**
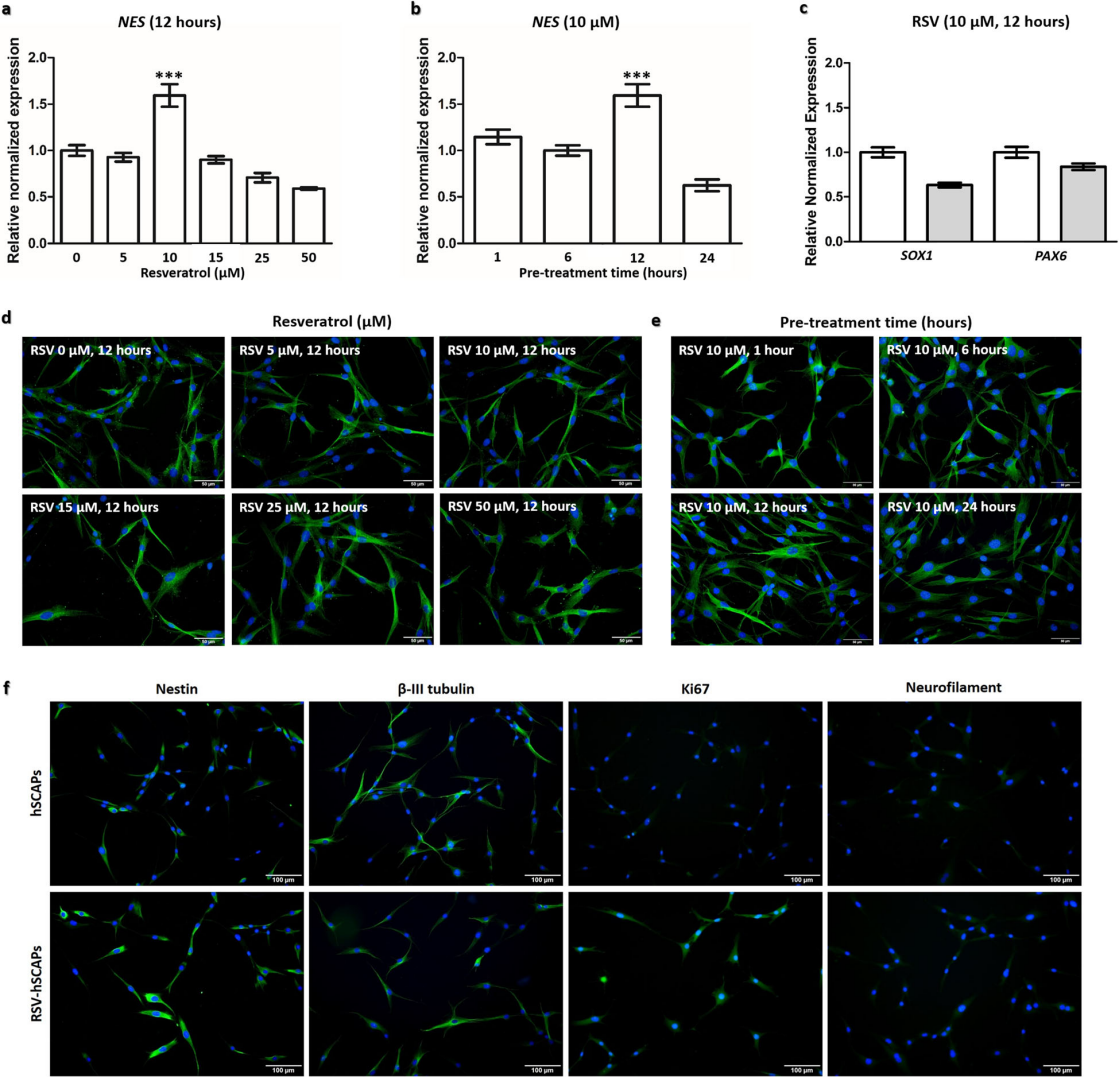
Optimal condition of resveratrol pre-treatment. **a** The expression of *NES* of resveratrol pre-treatment for 12 h ranging from 0 to 50 μM. The *NES* expression was significantly increased at 10 μM and dropped at 50 μM. **b** The *NES* expression of resveratrol pre-treatment at 10 μM for 1, 6, 12, and 24 h. The highest expression of *NES* was observed at 12 h and determined as an optimal condition. **c** Neural progenitor gene makers of resveratrol at 10 μM for 12 h condition were validated with *SOX1* and *PAX6*. Data were expressed as the mean ± SEM; *n* = 3, ***p < 0.001. **d** The cell morphology visualized by β-III tubulin staining of RSV-hSCAPs during 0–50 μM of resveratrol for 12 h. **e** The β-III tubulin profiling of RSV-hSCAPs at 10 μM for 1, 6, 12, and 24 h. **f** Nestin, β-III tubulin, Ki67, and NF immunostaining. These outcomes have strongly demonstrated that pre-treatment of resveratrol effectively induces neural progenitor gene marker expression but insufficiently triggers morphological change of the hSCAPs. Scale bars: **d**, **e** = 50 μm and **f** = 100 μm

To evaluate the effect of resveratrol on enhancing neuronal differentiation of hSCAPs, the cells were incubated with 0–50 μM of resveratrol for 12 h. We hypothesized that resveratrol pre-treatment would promote the expression of NPCs gene, *NES*. The qRT-PCR demonstrated that the *NES* expression significantly increased at 10 μM resveratrol pre-treatment, compared to control. However, the expression significantly decreased at 50 μM. (Fig. 3a). To determine optimal pre-treatment time, the hSCAPs treated with 10 μM resveratrol were investigated for *NES* expression at various incubation times: 1, 6, 12, and 24 h. The *NES* expression was significantly highest at 12 h and dropped at 24 h of pre-treatment time (Fig. 3b). The expression of other neural progenitor genes (*SOX1* and *PAX6*) of hSCAPs treated with 10 μM resveratrol for 12 h were not significantly different, compared to control (Fig. 3c). Moreover, the β-III tubulin immunofluorescence staining revealed that all of the resveratrol-treated hSCAPs ranging from 0 to 50 μM for 12 h (Fig. 3d) and 10 μM for 1, 6, 12, and 24 h of pre-treatment times (Fig. 3e) exhibited morphology as the typical fibroblast and spindle-like shape, which was similar to the control and the primary hSCAPs (Fig. 1a). Interestingly, the hSCAPs treated with 10 μM resveratrol for 12 h positively expressed Nestin, β-III tubulin, and Ki67 (proliferative marker) and negatively expressed neurofilament marker (Fig. 3f) suggesting that the resveratrol-treated hSCAPs have not yet differentiated into neuronal cells but resveratrol treatment activated neural progenitor gene expression (*NES*). Therefore, the pre-treatment of resveratrol at 10 μM for 12 h was determined as the optimal condition and referred to as RSV-hSCAPs.

### Neuronal induction

It was demonstrated that pre-treatment with 10 μM of resveratrol for 12 h actively promoted neural progenitor gene expression. To elucidate the effect of resveratrol on neuronal differentiation of hSCAPs, the cells were pre-treated with/without 10 μM of resveratrol for 12 h. We hypothesized that RSV-hSCAPs would differentiate into NPCs more than the hSCAPs. The hSCAPs and RSV-hSCAPs were consequently induced with 2 phases of neuronal induction medium.

**Fig. 4:**
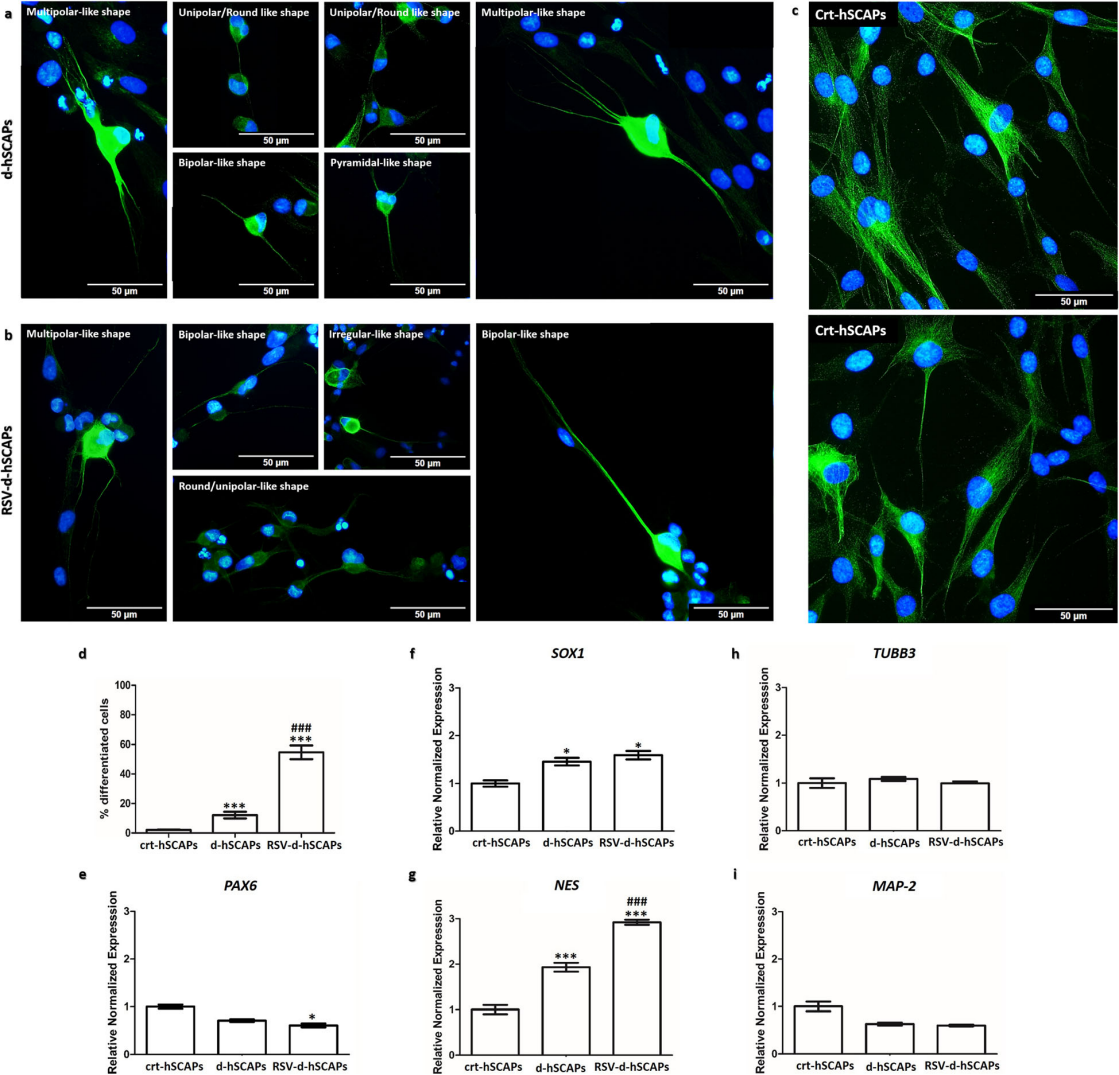
Neuronal induction. **a**–**c** The immunofluorescences profiling (β-III tubulin) of crt-hSCAPs, d-hSCAPs, and RSV-d-hSCAPs. These visualizations have revealed that a neuronal induction medium successfully promotes morphological change of d-hSCAPs and RSV-d-hSCAPs into neuronal-like cells, while the crt-hSCAPs still clearly exhibit as fibroblast-like cells. **d** The percentage of neuronal differentiation between d-hSCAPs and RSV-d-hSCAPs. Resveratrol pre-treatment efficiently enhances the percentage of differentiated cells from 12.11 ± 5.08 up to 54.71 ± 10.39. Data were expressed as the mean ± SEM; *n* = 5, ***p < 0.001 compared to crt-hSCAPs, ^###^*p <* 0.001 compared to d-hSCAPs. **e**–**i** The genes expression profiling (*PAX6*, *SOX1*, *NES*, *TUBB3*, and *MAP-2*) of crt-hSCAPs, d-hSCAPs, and RSV-d-hSCAPs. Resveratrol pre-treatment synergistically promotes the neural progenitor marker gene; the decreasing expression was expressed in *PAX6*. The increased expression was highly expressed in *SOX1* and *NES*, but not in*TUBB3* or *MAP-2*. Data were expressed as the mean ± SEM; *n* = 3, *p < 0.05, ***p < 0.001 compared to crt-hSCAPs, ^###^*p <* 0.001 compared to d-hSCAPs. Scale bars: **a**, **b**, and **c** = 100 μm

The hSCAPs and RSV-hSCAPs were differentiated (d-hSCAPs; Fig. 4a, and RSV-d-hSCAPs; Fig. 4b, respectively) as they showed neuronal-like appearances. Both d-hSCAPs’ and RSV-d-hSCAPs’ presented several types of neuronal-like morphology, such as round shape, unipolar shape, bipolar shape, multipolar shape, pyramidal shape, and irregular shape. On the other hand, the crt-hSCAPs, which were cultured in the medium without neuronal induction supplements, presented the typical fibroblast and spindle-like shape morphology (Fig. 4c), similar to the primary hSCAPs (Fig. 1a).

The percentage of neuronal differentiation of d-hSCAPs was 12.11 ± 5.08%. Interestingly, the neuronal differentiation of RSV-d-hSCAPs was significantly increased to 54.71 ± 10.39% (Fig. 4d). Moreover, the RSV-d-hSCAPs demonstrated the lowest expression of *PAX6* (Fig. 4e). In contrast, the highest expression of *NES* and *SOX1* was distinctly observed in RSV-d-hSCAPs, as compared to crt-hSCAPs and d-hSCAPs (Fig. 4f, and Fig. 4g). However, expressions of *MAP-2* and *TUBB3* genes, which represent late neurogenic and immature postmitotic neuron, were not significantly different between crt-hSCAPs, d-hSCAPs, and RSV-d-hSCAPs (Fig. 4h, and Fig. 4i).

### Characterization of neuronal cells

**Fig. 5:**
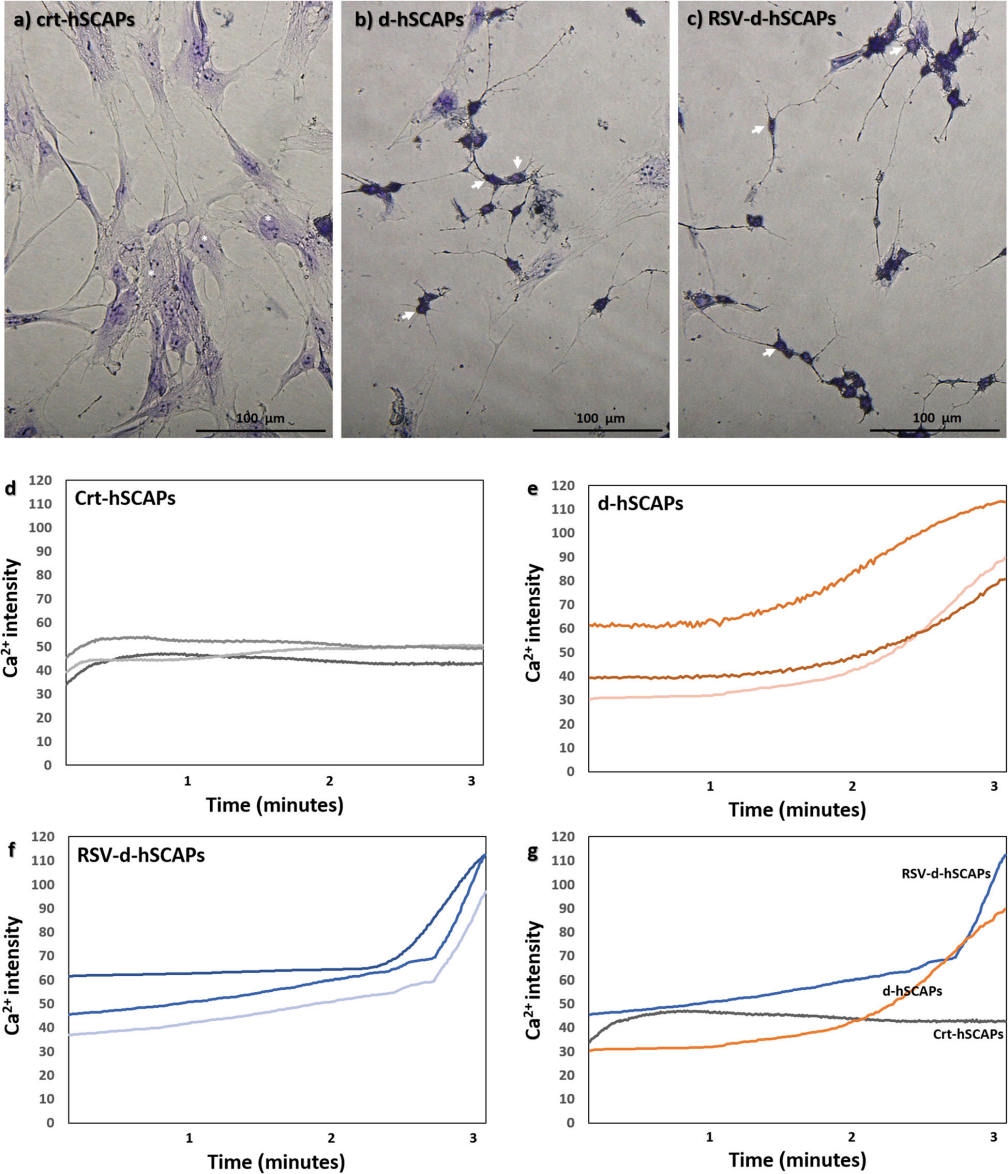
Characterization of neuronal cells. **a**–**c** Characterization of the neuronal cells with Cresyl violet staining. The differentiated cells from hSCAPs and RSV-hSCAPs revealed intense purple substance at cell body (white arrow), while the crt-hSCAPs showed the pale purple background of the nucleus (white asterisk) and dark violet of the nucleolus. Scale bars: **a**, **b**, and **c** = 100 μm. **d**–**f** The intracellular calcium oscillation of crt-hSCAPs, d-hSCAPs, and RSV-d-hSCAPs. The intensity of calcium has showed to be slightly increased and suddenly reached the highest peak in both d-hSCAPs and RSV-d-hSCAPs, while the hSCAPs exhibited steady patterns as a baseline intensity (*n* = 3). **g** The graph plotted between Ca^2+^ intensity and time (minutes) of crt-hSCAPs (gray line), d-hSCAPs (orange line), and RSV-d-hSCAPs (blue line)

Neuronal cell characteristics were validated by Cresyl violet staining. The Nissl granule as a prominence structure of neurons was observed. Interestingly, the differentiated cells from hSCAPs (Fig. 5b) and RSV-hSCAPs (Fig. 5c) exhibited the neuronal cells appearance and revealed intense purple substance at cell body (white arrow), while the crt-hSCAPs showed the pale purple background of the nucleus (white asterisk) and dark violet of the nucleolus with a typical fibroblast-like shape morphology (Fig. 5a). After neuronal induction, intracellular calcium oscillation was found in differentiated cells to characterize the neurotransmitter releasing activity of neuronal cells (Fig. 5g). Both calcium intensity of d-hSCAPs (Fig. 5e) and RSV-d-hSCAPs (Fig. 5f) showed slightly increased and suddenly reached the highest intensity after 2 min. On the other hand, the steady patterns as a baseline intensity were observed at the hSCAPs (Fig. 5d).

## Discussion

Mesenchymal stem cells of dental origin are being considered as a promising source for neurodegenerative therapies due to their self-renewal properties and multipotential differentiation [24]. The hSCAPs derived from a developing root represent a population of early stem cells [7]. Moreover, the hSCAPs have shown the ectomesenchyme origin with migratory neural crest stem cell derivative [25]. Recent studies have demonstrated the characteristics and capacity for multilineage differentiation of hSCAPs [26]. Among the various dental-origin stem cell populations, hSCAPs exhibited superior profiling, including multipotential differentiation, secretion of neurotrophic factors, and neurite outgrowth stimulation [27]. We characterized hSCAPs according to the minimal criteria of MSCs from the International Society for Cellular Therapy (ISCT) [8]. The isolated cells successfully grew on plastic culture vessels and exhibited the typical fibroblast and spindle-shaped morphologies, which represented plastic adherent abilities and the morphology of MSCs. The isolated cells were analyzed for the markers of MSCs. Flow cytometry demonstrated that the cells highly expressed CD73, CD90, CD105, and CD146 but negatively expressed CD34. The cells were able to form colonies that were visualized by Giemsa staining indicating their self-renewal ability. Moreover, to demonstrate multipotency properties, the isolated cells were induced with osteogenic and adipogenic induction media. The calcified nodule was visualized with Alizarin red, and lipid droplets were clearly stained with Oil Red O. Under neuronal induction, the isolated cells were efficiently differentiated into neuronal-like cells (Figs. 4a, and 5b). Finally, the isolated cells were positively stained with Nestin to reveal a migratory neural crest derivative of ectomesenchymal origin (Fig. 1d). Taken together, the results verified that the isolated cells established from human apical papilla tissue were MSCs, namely as hSCAPs.

Resveratrol is a non-flavonoid polyphenol compound with a stilbene structure obtained from various plants [18]. Resveratrol has 2 isomeric forms: *cis-*resveratrol and *trans*-resveratrol. Importantly, *trans-*isoform is a stable isoform and is the more predominant common active compound [28], while *cis*-isoform is unstable and the less common compound. However, the *trans*-resveratrol can be converted into *cis*-resveratrol after exposure with heat, UV radiation, or sunlight [29]. Therefore, we freshly prepared the resveratrol solution and maintained it in dark conditions for maximal efficiency. Previous studies have found that resveratrol did not have cytotoxicity on human dental pulp stem cells (hDPSCs) [15] or human bone marrow mesenchymal stem cells (hBM-MSCs) [20] at 0–50 μM concentrations for 12 h. Some studies reported that the cell viabilities of resveratrol-treated human umbilical cord-derived mesenchymal stem cells (hUC-MSCs) did not change at 0–50 μM for 24 h [21]. Despite present progress, there are no studies on the cell viability of hSCAPs with different concentrations of resveratrol. In this study, we first demonstrated the cellular toxicity effect of resveratrol on hSCAPs’ viability in order to select non-cellular toxic concentrations. The hSCAPs were treated with different concentrations of resveratrol (0, 5, 10, 15, 25, 50, and 100 μM) for 6, 12, and 24 h, and then the MTT assay was performed. We have clearly shown that the resveratrol treatment did not have a cytotoxic effect on the hSCAPs from 0 to 100 μM for 6 h, 0–50 μM for 12 h, and 0–15 μM for 24 h. Additionally, we were firstly reported the IC_50_ of resveratrol treatment on hSCAPs as 3380 mM (6 h), 1501 μM (12 h), and 73.33 μM (24 h). The cellular toxicity of resveratrol as explained by the hormetic dose-response effect lead to positive responses (associated with beneficial effects) at low concentrations and negative responses (associated with toxic effects) at high concentrations [30]. High concentrations of resveratrol promoted insufficient anti-oxidant defense system activities, induced disruption of mitochondrial membrane potential, and increased reactive oxygen species (ROS) production [31]. Furthermore, it was demonstrated that cell viability is dependent upon the duration of resveratrol supplementation. At longer exposure times, increased ROS secretions were observed, which lead to cell death [32]. Therefore, consistent with previous reports, we selected a non-cellular toxicity concentration of resveratrol from 0 to 50 μM for 12 h for further studies.

The optimal condition of resveratrol has effectively induced neuronal-associated gene expression and neuronal differentiation. Previous studies found that the expression of neural progenitor genes were the highest when hBM-MSCs were treated with resveratrol at 1 μM for 12 h [20]. Moreover, the treatment of the hDPSCs in 15 μM of resveratrol for 12 h was demonstrated as the optimal condition to induce the expression of *NES* [15], a gene encoded for Nestin protein which is highly expressed in neural progenitor cells in the subventricular zone of human brain [33]. In this study, we investigated resveratrol-induced neuronal differentiation of the hSCAPs. The characterized hSCAPs were incubated in the range of non-cellular toxicity concentrations of resveratrol (0–50 μM) for 12 h to achieve the highest expression of *NES*. Then, the concentration of resveratrol that drives the most *NES* expression was assessed at 1, 6, 12, and 24 h to determine the optimal pre-treatment time. We found that hSCAPs treated with 10 μM resveratrol for 12 h were the optimal condition to enhance *NES* expression, as revealed by qRT-PCR. To investigate an enrichment of neural progenitor cells genes expression, the RSV-hSCAPs were further accessed genes profiling with *SOX1* which was found in neural stem cells in the subventricular zone [33] and *PAX6* which uniformly expressed in early neuroectoderm cells [34]. However, the RT-qPCR demonstrated that the expression of these genes was not significantly different compared to control group and consistent with the hSCAPs which were already expressed certain level of *NES*, suggesting that the treatment 10 μM resveratrol for 12 h specifically enhanced *NES* expression. These findings correlated with previous studies to select optimal condition of resveratrol with *NES* expression [15, 20]. Moreover, the RSV-hSCAPs positively expressed Ki67, suggesting that resveratrol activated proliferative activity. Consistently, β-III tubulin staining showed that the cell morphology of RSV-hSCAPs was similar to primary hSCAPs, and NF was also negatively expressed (Fig. 3f). These results demonstrated that the RSV-hSCAPs were not differentiated into the neural progenitor cells. However, the optimal conditions of resveratrol which activated *NES* expression could be more effectively enhanced neuronal differentiation with further neuronal induction medium.

The resveratrol pre-treatment exerts concentration-specific biphasic responses involving stimulatory and inhibitory dual effects on neuronal progenitor gene expression. Recent studies show that a low concentration (≥ 10 μM) of resveratrol triggers the TrkA receptors, and consequently phosphorylated *SIRT1* and MAPK axis, and cascading downregulation increased CREB-TF (cAMP response element-binding protein transcription factor) involving the neural progenitor gene. On the other hand, higher concentrations (≥ 20 μM) of resveratrol inhibited the phosphorylation of TrkA and MAPK, signaling with a result low expression of *SIRT1*, and neural progenitor gene. Moreover, it also decreased the expression of the anti-apoptotic protein Bcl-2 with parallel increases of activated caspase-3 (hallmark of apoptosis) and p75^NTR^ (death receptor) [35]. Therefore, consistent with previous reports, experiments suggest that the maximal efficiency expression of *NES* depends on the optimal binding ability between resveratrol ligand and the TrkA receptor, which regrade to the high level of MAPK and *SIRT1* downregulation.

To further trigger neuronal differentiation, the hSCAPs and RSV-hSCAPs were synergistically cultured with a neuronal induction medium composed of specific chemical compounds and neurotrophic factors, including β-mercaptoethanol, DMSO, BHA, and bFGF, which served as extrinsic signaling factors for promoting morphological change and neuronal differentiation [36]. In previous studies, the hBM-MSCs and RSV-hBM-MSCs were differentiated into neuronal-like cells, which exhibited 2 dendrites with longer than 60 μm under a neuronal induction medium and positively expressed neurofilament protein. The high rate of neuronal differentiation was demonstrated from the RSV-d-hBM-MSCs [20]. Moreover, the differentiated cells derived from hDPSCs and RSV-hDPSCs (d-hDPSCs and RSV-d-hDPSCs) had significantly increased neuronal-specific marker genes, including *NES*, *MSI1*, and *NF-M*, which indicated that the RSV-d-hDPSCs were superior for neuronal differentiation profiling [15]. In this study, we have demonstrated that synergistically neuronal induction medium induced hSCAPs and RSV-hSCAPs into differentiated cells. Firstly, the differentiated cells derived from hSCAPs and RSV-hSCAPs (d-hSCAPs and RSV-d-hSCAPs) were exhibit neuronal-like morphology in several types, including round shape, unipolar shape, bipolar shape, multipolar shape, irregular shape, and pyramidal shape (Figs. 4a, b) with β-III tubulin positive staining, whereas the crt-hSCAPs, which were cultured with a basal medium, presented a flattened shaped (Fig. 4c) as did the primary hSCAPs. Secondly, our study also demonstrated that both d-hSCAPs and RSV-d-hSCAPs revealed intense purple granule of Nissl substance, a hallmark of neurons [37], at cell body, while the crt-hSCAPs showed the dark violet of nucleolus in pale purple background of the nucleus, suggesting that the d-hSCAPs and RSV-d-hSCAPs were characterized as neuronal cells. Interestingly, resveratrol pre-treatment effectively enhances neuronal differentiation. The percentage of differentiated cells was significantly increased from 12.11 ± 5.08 (d-hSCAPs) to up to 54.71 ± 10.39 (RSV-d-hSCAPs).

Neuronal-specific marker genes expression was evaluated to confirm the neural progenitor cells. A recent study demonstrated that the neural progenitor cells derived from hDPSCs were insufficiently expressed of *PAX6*. Taken together, highly expressed *NES* and *SOX1* and weakly expressed *PAX6* profiling are used as early stage markers of neuronal differentiation [38]. Moreover, a previous study has shown the neuronal differentiation potential of hSCAPs. The differentiated cells derived from hSCAPs exhibited neuronal-like cell morphology under long-term neuronal induction for 5 weeks. However, the qRT-PCR has revealed that the differentiated cells were highly expressed *NSE* and weakly detected in *TUBB3* and *NF-M*. This evidence suggests that the hSCAPs are more restricted and committed in their neuronal differentiation at an early stage [38]. Our results provided 2 sequential phases of neuronal induction; the differentiated cells presented that the increasing expression was only observed in *NES* and *SOX1* but not in *PAX6* and *MAP-2* or *TUBB3* in d-hSCAPs, as revealed by qRT-PCR. Our results were consistent with the previous study, which strongly confirmed that our differentiated cells were characterized as neural progenitor cells. Importantly, we have found that the synergistic pre-treatment resveratrol with neuronal induction medium triggered differentiation into neural progenitor-like cells with specific expressions of *NES* and *SOX1* and showed a higher percentage of neuronal differentiation than d-hSCAPs by 4 times.

Recent approaches have been defined parameters to evaluate in vitro induced neurons [39]. First are structural appearances that included cytoskeletal proteins. The differentiated cells exhibited a neuronal shape which positively detected neural cytoskeletal proteins such as β-III tubulin and neurofilament [12, 13, 20] or neuronal cell-specific markers such as Nestin, CD133, glial fibrillary acid protein (GFAP), MAP-2, enolase, and synaptophysin [11, 15, 20, 21]. Second are functional characteristics involving functional neuronal networks, intercellular communication [40, 41], and intracellular signaling cascades [12, 23]. Calcium ions are internalized into neurons to modulate vesicular neurotransmitter releasing [42]. Intracellular calcium activity has been used to represent neuronal activity as it closely correlated with electrical activity recorded from whole-cell patch-clamp [43]. In this study, we have observed intracellular calcium oscillation to prove functionality of differentiated cells. We use Fluo-3 as an indicator to detect dynamics of intracellular calcium signaling during neurotransmitter transmission [44]. Previously, the neuronal cell-derived human cord blood mesenchymal stem cells which are triggered by the combination between resveratrol and nerve growth factor for 4 days demonstrated the increasing of intracellular Ca^2+^ level in a time-dependent manner [12]. Our study showed a slightly increasing pattern of calcium intensity in d-hSCAPs and RSV-d-hSCAPs, while the steady patterns as a baseline intensity were observed only in hSCAPs suggesting that the d-hSCAPs and RSV-d-hSCAPs were confirmed as functional neuronal cells.

The application of resveratrol to mesenchymal stem cell-based regenerative medicine has been demonstrated in various in vitro bioactivities, including self-renewal, multipotency [45], senescence [21], cell aging [46], osteogenic differentiation [47], adipogenic differentiation [48], and neuronal differentiation [12]. Importantly, in vitro models have the potential to demonstrate insight into cellular and molecular mechanisms. Moreover, reduction of animal use and increasing time- and cost-effectiveness were undertaken on in vitro model [49]. The potential effects of resveratrol are triggered by *SIRT1* activation. The *SIRT1* acts as the central modulator of bioactivities signaling pathways [50]. Resveratrol indirectly activates *SIRT1* by increasing intracellular cAMP following the inhibition of cAMP-dependent phosphodiesterase (PDE) [51]. *SIRT1* strongly promotes neuronal differentiation through PKA/GSK3-β/β-catenin and PKA/ERK1/2 axis [12]. *SIRT1* also plays important roles in controlling microtubule dynamics and neurite outgrowth stimulation during axon elongation by deacetylating AKT [52]. Taken together, previous studies confirm the potential effect of resveratrol, through *SIRT1* activation, on neuronal differentiation by changing the structural features of the cell into neuronal phenotype.

Moreover, resveratrol has an efficiently therapeutic effect on in vivo models, including enhancing liver regeneration [53] and cardiogenic differentiation in cardiomyopathy [54]. Interestingly, oral administration of *trans-*resveratrol 20 mg/kg body weight for 45 days into aged rat has recovered the number of newly generated neurons in the hippocampus with positive Nissl substance and BrdU (proliferative marker) labeling [35]. Additionally, resveratrol treatment improved learning and memory and enhanced neurogenesis in Alzheimer’s disease (AD) mouse model [55]. However, the transplantation of resveratrol-treated hSCAPs or NPCs derived from hSCAPs into animal model of neurodegenerative disease needs to be performed in future studies to reflect the correlation of potential effects of resveratrol on neuronal differentiation between in vitro and in vivo models.

Taken together, we have demonstrated that resveratrol serves as an effective enhancer of neuronal differentiation by promoting neural progenitor gene expression in the hSCAPs and that the RSV-hSCAPs are more differentiated into neuronal-like cells at the early stage than at the late stage in a neuronal induction medium. These results suggest that a resveratrol pre-treatment of MSCs may be an effective alternative approach for neurodegenerative disease.

## Conclusion

This study demonstrated the capacity of hSCAPs for neuronal differentiation and that pre-treatment with resveratrol efficiently induces neural progenitor marker gene expression, which synergistically enhances neural progenitor-like cell induction within a neuronal induction medium. Thus, these findings suggest the alternative of using hSCAPs and the potential of resveratrol treatment as a stem cell-based therapy, for further transplantation in the treatment of neurodegenerative disease.

## Data Availability

Not applicable

## Abbreviations

A570-A690: Absorbance at 570 nm minus 690 nm
AD: Alzheimer’s disease
AKT: Protein kinase B
bFGF: Basic fibroblast growth factor
BHA: Butylated hydroxyanisole
cAMP: Cyclic adenosine monophosphate
CD: Cluster of differentiation
CNS: Central nervous system
Crt-hSCAPs: Control hSCAPs
d-hBM-MSCs: Differentiated cell-derived human bone marrow mesenchymal stem cells
d-hDPSCs: Differentiated cell-derived human dental pulp stem cells
d-hSCAPs: Differentiated cell-derived human stem cells from apical papilla
DMEM/F-12: Dulbecco’s Modified Eagle Medium: Nutrient Mixture F^-12^ (Ham)
DMSO: Dimethyl sulfoxide
DSCs: Dental-derived stem cells
ERK1/2: Extracellular signaling regulated kinase1/2
FBS: Fetal bovine serum
*GAPDH*: Glyceraldehyde 3-phosphate dehydrogenase
GFAP: Glial fibrillary acid protein
GSK3-β: Glycogen synthase kinase 3-β
hBM-MSCs: Human bone marrow-derived mesenchymal stem cells
hDPSCs: Human dental pulp stem cells
hSCAPs: Human stem cells from apical papilla
hUC-MSCs: Human umbilical cord-derived mesenchymal stem cells
IBMX: 3-Isobutyl-1-methylxanthine
IC_50_: 50% inhibitory concentration
ISCT: International Society for Cellular Therapy
*MAP-2*: Microtubule associated protein-2
MSCs: Mesenchymal stem cells
MTT: Methylthiazolyldiphenyl-tetrazolium bromide
NF: Neurofilaments
*NF-M*: Neurofilament medium-type
NPCs: Neural progenitor cells
*NES*: Human gene encoded for Nestin protein
PDE: Phosphodiesterase
PKA: Protein kinase A
qRT-PCR: Quantitative real-time polymerase chain reaction
RSV: Resveratrol
RSV-d-hBM-MSCs: Differentiated cell-derived resveratrol pre-treated hBM-MSCs
RSV-d-hDPSCs: Differentiated cell-derived resveratrol pre-treated hDPSCs
RSV-d-hSCAPs: Differentiated cell-derived resveratrol pre-treated hSCAPs
RSV-hSCAPs: Resveratrol pre-treated hSCAPs at optimal condition
SEM: Standard error of mean
*SIRT1*: Sirtuin 1
*TrkA*: Tyrosine kinase receptor type 1
αMEM: Alpha Minimum Essential Medium
